# Reaction Kinetics in the Vermicomposting Process of Peach Waste

**DOI:** 10.3390/life12091290

**Published:** 2022-08-23

**Authors:** Lorena De Medina-Salas, Eduardo Castillo-González, Mario Rafael Giraldi-Díaz, Berenice Blanco-Pérez

**Affiliations:** 1 Facultad de Ciencias Químicas, Universidad Veracruzana, Circuito Gonzalo Aguirre Beltrán s/n, Zona Universitaria, Xalapa 91040, Veracruz, Mexico; 2 Facultad de Ingeniería Civil, Universidad Veracruzana, Circuito Gonzalo Aguirre Beltrán s/n, Zona Universitaria, Xalapa 91040, Veracruz, Mexico

**Keywords:** vermicomposting, peach waste, kinetics models, reaction rate constant, order reaction

## Abstract

Peach is a fruit cultivated in temperate regions and its use generates waste composed of seeds and skin. Inadequate disposal of this waste generates an environmental impact; therefore, an alternative is to apply a vermicomposting degradation process. In this research, these four laboratory-scale reactors were used: RC (no earthworms), R1, R2, and R3 (50 earthworms each) to get mixtures in the following proportions of peach waste and load material (vegetable waste and eggshell): RC (50%-50%), R1 (50%-50%), R2 (60%-40%), and R3 (40%-60%). In addition, during this process, physicochemical parameters were analyzed (temperature, pH, humidity, total organic carbon (TOC), total nitrogen (TN), and carbon/nitrogen ratio (C/N)). For each mixture, the reaction order and rate constants were determined using mathematical models. After analysis of the reaction kinetics, the results showed that zero- and first-order reactions were best suited for the degradation of this waste in the vermicomposting process. The highest rates of degradation in the mixtures were for RC and R1, which means faster completion of the process, and consequently, smaller dimensions of the facilities necessary for vermicomposting. Thus, this research provides important information for the design of reactors that use similar substrates.

## 1. Introduction

Peach (*Prunus persica*) is a round fruit that measures 5 to 7.5 cm in diameter. It is yellow with reddish tinges and has a velvet-like texture and a seed in the center. Along with apples, strawberries, pears, plums, and cherries, it belongs to the *Rosaceae* family. Similar to almonds, its seeds are used as an oil substitute in the field of cosmetics [1].

In the 2020/21 cycle, world peach production was 21,029,000 metric tons according to data obtained from the United States Department of Agriculture (USDA) Foreign Agricultural Service. China predominates with 14,500,000 metric tons (68.95%), followed by the European Union with 3,475,000 metric tons (16.52%), and Turkey with 870,000 metric tons (4.14%) [2].

Peach waste is composed of seeds and skin [3]. Its seeds contain 50% oil [4], 27.5% protein, and nutritional properties owing to the presence of unsaturated fats with high oleic (58%), linoleic (32%), and palmitic acid (8%) content [5].

To dispose of fruit waste, there are several alternatives, including landfilling and open burning. If there is no control, the use of these alternatives can cause various environmental impacts, such as generation of unpleasant odors and release of greenhouse gases that contribute to global warming and atmospheric pollution, which may affect human health [6].

However, there are different techniques to valorize organic waste, such as composting, vermicomposting, anaerobic digestion, and entomoremediation using insect larvae, i.e., black soldier fly larvae [7,8,9], among others.

Composting and vermicomposting of organic waste have been considered economically viable and sustainable waste management technologies [10]. Composting is an aerobic process that, under proper temperature and humidity conditions, results in stable materials that can be used for soil treatment in agriculture [11]. Vermicomposting is a practice involving the addition of earthworms to the composting process. Both techniques are inexpensive and improve soils due to their macro- and micronutrients [12].

Vermicomposting allows organic nutrient sources for crops to be obtained in less time, which is nutritionally (nitrogen, potassium, phosphorus, and calcium), physically, and biochemically efficient [13,14] as a soil conditioner [15]. It is also one of the most feasible and environmentally friendly techniques for the bioconversion of industrial wastes/sludges into useful and high-quality vermicompost [16]

For vermicomposting to be implemented on an industrial scale, it is necessary to design reactors and treatment facilities properly. Therefore, it is fundamental to understand the reaction kinetics that occur during these processes, considering that high degradation rates lead to lower construction and operational costs for composting and vermicomposting plants [17,18,19].

The reaction kinetics of multiple types of waste going through the composting process have been studied [19,20,21,22,23,24,25]. The published studies refer to composting processes for different organic wastes and only a few of them correspond to vermicomposting [26,27,28,29]. However, after conducting an exhaustive literature search, no research related to reaction kinetics was found for vermicomposting of peach waste.

Thus, this research aimed to analyze the reaction kinetics behavior during the biodegradation of peach waste in different mixtures through the vermicomposting process to determine reaction order and kinetics coefficients associated with the process, setting the hypothesis that the kinetics of the vermicomposting process for peach waste fits a combined zero-order and first-order reaction.

## 2. Materials and Methods

### 2.1. Substrate

The substrate was made up of 10 kg of peach waste (seeds and skin) to which load material was added; the latter included vegetable waste, comprised of chard, celery, broccoli, watercress, cabbage, spinach, and lettuce (10 kg), as well as eggshells (1 kg) to balance nutrients. Waste was collected from local markets and bakeries and finely chopped into pieces ranging between 1 and 5 cm to ease the degradation [30,31]. The eggshells were washed and sun-dried for 24 h [32] and crushed to a size of 0.5 mm.

### 2.2. Vermicomposting Process

Before the vermicomposting process, the peach waste and load material were subjected to an initial pre-composting degradation using two plastic containers, each with a capacity of 11.4 L. The first one contained the peach waste only and the second, the load material. Both containers were covered with a 5 mm mosquito net to allow gaseous exchange and avoid potential insect or other undesirable animal infestation [16,33]. The bottom of both containers was perforated to filter out leachate. The holes were 9 mm in diameter and were placed at a 2% slope to ease drainage.

The peach waste was pre-composted for 30 days and humidified daily by being sprayed with water to maintain the humidity parameter within the proper range for the process. To control this parameter, the fist test was used, as it allows the maintenance of the correct amount of humidity [34]. Load material was pre-composted 72 h before the beginning of the pre-composting process, as the degradation rate was high due to its highwater content.

The vermicomposting process was performed using four plastic containers measuring 32 cm in length, 9 cm in width, and 20 cm in height, making up a volume of 5.76 L. The proportions used in each pre-composted mixture are shown in Table 1.

As was the case in the pre-composting process, the containers were perforated at the bottom and covered with a fine mesh. The first reactor (RC) was a control; therefore, no earthworms were added. The rest of the reactors (R1, R2, and R3) received 50 *Eisenia fetida* earthworms each because it is one of the most common species used in vermicomposting [35,36,37,38]. For the experiment, adult earthworms with an initial size of 5–6 cm and an average weight of 1 g were obtained from a vermicomposting plant.

During the first five days of the process, the reactors were kept in repose so the earthworms could adapt to their new environment. The reactors were mixed daily to guarantee the vermicompost was properly aerated and were sprayed with water every third day to maintain the required humidity level [39]. During this process, the earthworms fed off the available organic matter.

### 2.3. Analytical Methods

During the vermicomposting process, the following physicochemical parameters were determined in the laboratory using local norms and by triplicate: temperature, pH, humidity, total organic carbon (TOC), total nitrogen (TN), and carbon/nitrogen ratio (C/N) [40].

### 2.4. Statistical Data Analysis

For the statistical analysis, weekly TOC concentration data for each of the reactors were used. The coefficient of determination (R^2^) was determined to identify reaction orders in each mixture. The equation associated with each reaction order was obtained to determine kinetics coefficients. Data analysis was performed using Microsoft Excel version 16.61.

### 2.5. Kinetics Models

For the kinetics analysis of this research, figures corresponding to the TOC values were made. These data were obtained weekly for each reactor RC, R1, R2, and R3. Subsequently, reaction orders were determined and analyzed using the following equations [41,42,43,44]:

#### 2.5.1. Zero-Order Reactions

Equation (1) was used for zero-order reactions

(1) $$-\frac{d\left(\mathrm{TOC}\right)}{\mathrm{dt}}=\;k$$

where

$$-\frac{d\left(\mathrm{TOC}\right)}{\mathrm{dt}}=\;\mathrm{rate}\;\mathrm{of}\;\mathrm{TOC}\;\mathrm{change}\;\mathrm{with}\;\mathrm{respect}\;\mathrm{to}\;\mathrm{time}\left[\frac{\mathrm{Percentage}}{\mathrm{Time}}\right]$$

k = reaction rate constant $\left[t^{-1}\right]$

Linearizing led to Equation (2):

(2) $$C\;=-\mathrm{kt}\;+{\;C}_{o}$$

where

C = TOC concentration at any time t [percentage]

C_0_ = initial TOC concentration for t = 0 [percentage]

t = time it takes for the reaction to take place [t]

k = reaction rate constant $\left[t^{-1}\right]$

#### 2.5.2. First-Order Reactions

Equation (3) was used for first-order reactions:

(3) $$-\frac{\mathrm{dC}}{\mathrm{dt}}=k{\left(C\right)}^{1}$$

where

$$-\frac{\mathrm{dC}}{\mathrm{dt}}=\mathrm{rate}\;\mathrm{of}\;\mathrm{TOC}\;\mathrm{change}\;\mathrm{with}\;\mathrm{respect}\;\mathrm{to}\;\mathrm{time}\;\left[\frac{\mathrm{percentage}}{\mathrm{time}}\right]\;$$

k = reaction rate constant $\left[t^{-1}\right]$

C = TOC concentration at any time t [percentage]

Linearizing the previous equation led to Equation (4):

(4) $$\ln\left(\frac{C_{0}}{C}\right)=\mathrm{kt}$$

where

C_0_ = initial TOC concentration for t = 0 [percentage]

t = time it takes for the reaction to take place [t]

k = reaction rate constant $\left[t^{-1}\right]$

C = TOC concentration at any time t [percentage]

#### 2.5.3. Second-Order Reactions

Equation (5) was used for second-order reactions:

(5) $$-\frac{\mathrm{dC}}{\mathrm{dt}}=\;k{\left(C\right)}^{2}$$

where

$$-\frac{\mathrm{dC}}{\mathrm{dt}}=\mathrm{rate}\;\mathrm{of}\;\mathrm{TOC}\;\mathrm{change}\;\mathrm{with}\;\mathrm{respect}\;\mathrm{to}\;\mathrm{time}\left[\frac{\mathrm{percentage}}{\mathrm{time}}\right]$$

k = reaction rate constant $\left[d^{-1}\right]$

C = TOC concentration at any time t [percentage]

When linearized it led to Equation (6):

(6) $$\frac{1}{C}=\mathrm{kt}\;+\frac{1}{C_{0}}$$

where

C_0_ = initial TOC concentration for t = 0 [percentage]

C = TOC concentration at any time t [percentage]

k = reaction rate constant $\left[d^{-1}\right]$

t = time it takes for the reaction to take place [t]

## 3. Results and Discussion

### 3.1. Physicochemical Parameters in Vermicomposting

The temperature for all four reactors was kept between 18 and 24 °C. Multiple authors have reported that the optimal temperature for earthworm development is within the 15–28 °C range [45,46]. The initial pH for all four mixtures was between 6 and 6.5. In the beginning, pH decreased to values as low as 5.5 due to the generation of organic acids caused by the organic matter degradation [47]. At the end of the process, the pH range remained within neutral values between 6 and 7.5, which indicates waste stabilization. The humidity range during the process was maintained between 62 and 83.87%. Huang et al., (2016) and Othman et al., (2012) also reported values from 60 to 80% [45,48].

Organic matter concentration values for all four mixtures at the beginning of the process were 87.21%, 86.12%, 84.86%, and 81.74%, whereas final concentrations were found to be 48.34%, 47.91%, 45.02%, and 43.69% for reactors RC, R1, R2, and R3, respectively. The decrease in organic matter concentration was due to the degradation caused by the organisms that used carbon as an energy source. Chang et al., (2016) found a decrease in organic matter concentrations for pig manure and rice straw waste composting processes due to mineralization and carbon loss through carbon dioxide [49].

Total nitrogen content for reactors RC, R1, R2, and R3 were 1.77%, 1.46%, 1.21%, and 1.23%, while final values were found to be 2.45%, 3.06%, 3.71%, and 3.85%, respectively. An increase in nitrogen concentration could be observed in every reactor, coinciding with the consulted literature [50].

For the C/N ratio, the following values were obtained at the beginning of the process for reactors RC, R1, R2, and R3: 28.58, 34.21, 40.68, and 36.47, respectively. These values were adequate for the beginning of the vermicomposting process [51]. The final values of the C/N ratio for the reactors were 11.44, 9.08, 7.04, and 6.58. Several authors have reported that the C/N values at the end of the vermicomposting process must be less than 20 [52,53,54].

Table 2 shows the physicochemical values of pH, organic matter, total nitrogen, and C/N ratio obtained at the end of the vermicomposting process. This reveals that at 63 days a stabilized product was obtained and its quality complied with the Mexican Standard NMX-FF-109-SCFI-2007 [40] per the following values: pH 5.5–8.5, organic matter 20–50%, total nitrogen 1–4%, and a C/N ratio less than 20. These values confirmed that the vermicomposting process had reached its end.

### 3.2. Reaction Kinetics Analysis

Table 3 shows TOC behavior during the period of the vermicomposting process. Concentrations in all four reactors decreased consecutively throughout each of the seven weeks (63 days) due to the mineralization of organic matter to CO_2_ carried out by the earthworms and microorganisms [46].

**Figure 1:**
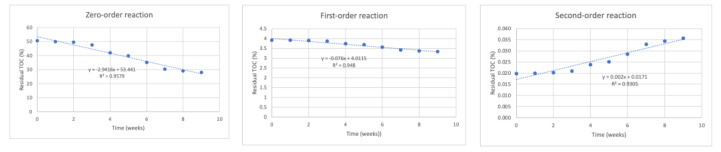
Application of zero-order, first-order, and second-order reaction models for the degradation of peach waste using remaining TOC concentration data for reactor RC (50% peach waste–50% load material, no earthworms).

**Figure 2:**
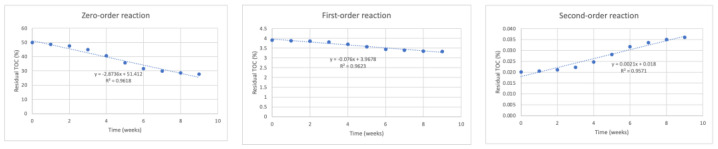
Application of zero-order, first-order, and second-order reaction models for the degradation of peach waste using remaining TOC concentration data for reactor R1 (50% peach waste–50% load material, with 50 earthworms).

**Figure 3:**
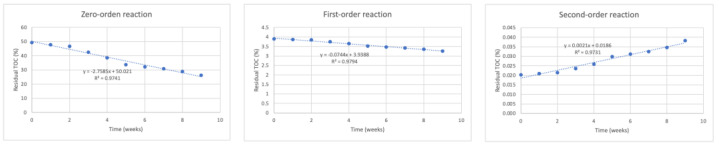
Application of zero-order, first-order, and second-order reaction models for the degradation of peach waste using remaining TOC concentration data for reactor R2 (60% peach waste–40% load material, with 50 earthworms).

**Figure 4:**
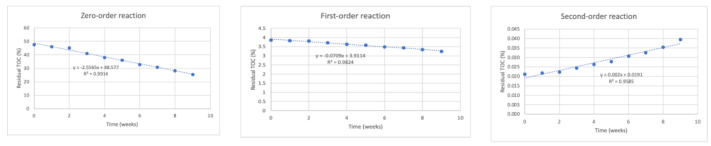
Application of zero-order, first-order, and second-order reaction models for the degradation of peach waste using remaining TOC concentration data for reactor R3 (40% peach waste–60% load material, with 50 earthworms).

Figure 1, Figure 2, Figure 3 and Figure 4 show degradation behavior in the mixtures, in terms of TOC, for reactors RC, R1, R2, and R3, respectively, for zero-order, first-order, and second-order reactions.

Table 4 shows the values of R^2^ and k obtained from the aforementioned figures of zero-order, first-order, and second-order reactions in all four reactors.

This table allows the observation of concentration behaviors in the mixtures of peach waste fitted to zero-order and first-order reactions, as the R^2^ values were approximately 1.

Results coincided with other studies with carbon mineralization rates and we found that the process followed a combined zero-order and first-order kinetics reaction model for mixtures of organic waste with sludges from wastewater treatment plants, animal manures, urban and industrial waste, and vegetables from composting processes [55].

Abu and Al-Widyan also found high R^2^ values of 0.99, 0.98, and 0.88 for grain dust, grain dust with coffee processing waste, and coffee-processing waste, respectively [21]. On the other hand, Rastogi et al. obtained R^2^ values from 0.743 to 0.992 for municipal solid waste composting [24].

Zero-order reactions are characterized because the change in concentration with respect to time is independent of concentration [42,43], indicating that there is a great affinity between the substrate and the organisms responsible for its degradation. The higher the complexity of the organic compounds, the higher the reaction order. However, according to Petric et al., many researchers found that the organic matter degradation follows first-order kinetics [19] because it is widely applied in heterogeneous substrates, as in the case of the present research.

For first-order reactions, the reactors RC and R1 showed the fastest reaction rate for the biodegradation of the peach waste mixtures, as they obtained the highest value (k = 0.0760 d^−1^) in comparison to R2 (k = 0.0744 d^−1^) and R3 (k = 0.0709 d^−1^). RC and R1 had the same percentage of waste mixtures and load material (50–50%), which implies the reaction rate was the same in the vermicomposting process as in the control sample that did not contain earthworms (composting). These results coincided with the information reported by Fatemeh et al. because the mixture with the fastest reaction rate was the one that contained 50% palm oil mill effluent (POME) mixed with palm-pressed fiber [28].

The k values in all four mixtures were found to be within the 0.020–1.13 d^−1^ range, as reported by Kumar et al. [23]. Additionally, Tosun et al. reported ranges for reaction rate constants of 0.087–0.236 d^−1^ for rose flower waste and the organic fraction of municipal solid waste, fitting to a first-order kinetics model [20]: 0.005–0.1 d^−1^ for municipal solid waste and yard waste (food waste, mixed paper, leaves, branches, grass clippings) [56]; and 0.043–0.082 d^−1^ for municipal solid waste [57].

Similarly, k values obtained were higher than those reported in other studies: 0.0204 d^−1^ for the composting of biodegradable polymers [25]; 0.0195–0.0523 d^−1^ for the degradation of POME, also for first-order kinetics [28]; 0.0015–0.0055 d^−1^ for a variety of agro-industrial waste, applying first-order kinetics [21]; 0.01–0.02 d^−1^ for kitchen waste, pruned elm tree branches, and sheep manure [58]; and 0.044–0.045 d^−1^ for sludge sewage with lignocellulosic waste (wood chips, wheat straw, leaves) [59].

The k values obtained in all four mixtures were less than those reported in another vermicomposting study that resulted in a range of 0.12–0.59, with different substrates of cow manure waste and a filter cake made from sugarcane [29].

Table 5 shows the k values of different substrates reported in the literature. The variability of k values reported by several authors is due to the heterogeneous composition of the biodegradable waste, taking into consideration that a proper nutrient balance allows for a faster degradation rate. The trend in the degradation of organic waste, through the vermicomposting process, is to use balanced substrates enriched by adding nutrients. As shown in Table 5, researchers used an organic waste mixed with other substances to balance nutrients and facilitate degradation, while in this research, vegetable waste and eggshells were used for the same purpose and similar results were obtained. The advantage of making mixtures of a substrate with others that provide nutrients is to facilitate the degradation process because it is achieving a greater affinity between the substrate and the organisms responsible for the degradation, which is closely related to the order reaction.

## 4. Conclusions

This study analyzed kinetics reaction orders in the vermicomposting process of four different mixtures of peach waste and load material. According to the proposed hypothesis, it was determined that the degradation fit a combined zero-order and first-order kinetics, which revealed that earthworms and microorganisms had a high affinity for this waste.

Additionally, mixtures RC and R1, made up of 50% peach waste and 50% load material with and without earthworms, had the fastest reaction rates due to the highest k value. Therefore, these mixtures were the most adequate for peach waste degradation.

The results obtained from the kinetics coefficients can be applied to the design and operation of vermicomposting facilities. For future research, this study can be a referent for the exploration of new kinetics models that expand the knowledge of mechanisms under which the degradation processes of organic matter occur, in order to face the challenges of proper waste management.

## Figures and Tables

**Table 1:** Amount of peach and vegetable waste, eggshells, and earthworms in each waste mixture in the four reactors for the vermicomposting process.

Reactor	Peach Waste (kg)	Vegetable Waste (kg)	Eggshells (kg)	Peach Waste and Load Material Percentages	Number of Earthworms
RC	1.00	0.75	0.25	50-50%	0
R1	1.00	0.75	0.25	50-50%	50
R2	1.20	0.60	0.20	60-40%	50
R3	0.80	0.90	0.30	40-60%	50

**Table 2:** Results of the physicochemical parameters at the end of the vermicomposting process after 63 days for the four reactors of the experiment and the Local Standard comparison. Values are presented as mean and standard deviation (*n* = 3).

Reactor	pH	Organic Matter	N	C/N
RC	7.00 ± 0.18	48.34 ± 1.64	2.45 ± 0.04	11.44 ± 0.51
R1	6.95 ± 0.12	47.91 ± 1.58	3.06 ± 0.06	9.08 ± 0.55
R2	7.50 ± 0.20	45.02 ± 1.99	3.71 ± 0.06	7.04 ± 1.01
R3	6.00 ± 0.17	43.69 ± 1.10	3.85 ± 0.07	6.58 ± 1.03
Mexican Standard [40]	5.5–8.5	20–50%	1–4%	Less than 20

**Table 3:** Results of remaining TOC concentration during the 9 weeks of the vermicomposting process for each reactor.

Vermicomposting Process (days)	Reactor			
	TOC in RC (%)	TOC in R1 (%)	TOC in R2 (%)	TOC in R3 (%)
0	50.59 ± 1.72	49.95 ± 0.91	49.22 ± 1.68	47.41 ± 0.75
7	50.01 ± 1.40	48.66 ± 1.23	47.76 ± 1.25	46.05 ± 1.22
14	49.99 ± 1.58	47.45 ± 0.72	46.66 ± 1.55	45.10 ± 0.86
21	47.62 ± 1.19	44.97 ± 0.82	42.37 ± 1.02	41.06 ± 1.00
28	41.95 ± 1.55	40.59 ± 1.06	38.58 ± 1.36	38.00 ± 0.56
35	39.89 ± 1.28	35.60 ± 0.43	33.57 ± 1.01	36.03 ± 1.11
42	35.11 ± 0.91	31.49 ± 0.70	32.10 ± 0.77	32.66 ± 0.59
49	30.32 ± 0.87	29.80 ± 0.74	30.85 ± 0.98	30.85 ± 0.89
56	29.01 ± 0.65	28.51 ± 0.82	28.86 ± 0.56	28.23 ± 0.97
63	28.04 ± 0.76	27.79 ± 0.55	26.11 ± 1.03	25.34 ± 0.49

**Table 4:** Values of coefficient of determination (R^2^) and reaction rate constants (k) in all four reactors according to zero-order, first-order, and second-order reaction models.

Reactor	Zero-Order		First-Order		Second-Order	
	R^2^	k (d^−1^)	R^2^	k (d^−1^)	R^2^	k (d^−1^)
RC	0.9579	2.9416	0.9480	0.0760	0.9305	0.0020
R1	0.9618	2.8736	0.9623	0.0760	0.9571	0.0021
R2	0.9741	2.7585	0.9794	0.0744	0.9731	0.0021
R3	0.9914	2.5565	0.9824	0.0709	0.9585	0.0020

**Table 5:** Reaction rate constants (k) for different waste types.

S. No	Main Waste Type	Secondary Waste Type	k Values (d^−1^)	References	
1	Peach waste	Vegetable waste and eggshell	0.0709–0.0760	This research (2022)	-
2	Biodegradable polymers	Organic fraction MSW	0.0204	Rossetti et al., (2021)	[25]
3	Poly (lactic acid) (PLA)	Food waste compost	0.020–1.13	Kumar et al., (2021)	[23]
4	Rose composting	Organic fraction MSW	0.087–0.236	Tosun et al., (2008)	[20]
5	Palm oil mill effluent	Palm-pressed fiber	0.0195–0.0523	Fatemeh et al., (2017)	[28]
6	Kitchen waste (mixtures), pruned elm tree branches and sheep manure	Not applicable	0.01–0.02	Ebrahimzadeh et al., (2017)	[58]
7	Agro-industrial waste (mixtures) olive milling waste, grain dust, coffee processing wastes	Not applicable	0.0015–0.0055	Abu & Al-Widyan (2016)	[21]
8	Sewage sludge	Lignocellulosic waste (wood chips, wheat straw, leaves)	0.044–0.045	Kulikowska (2016)	[59]
9	Municipal solid waste	Not applicable	0.043–0.082	Baptista et al., (2010)	[57]
10	Cow manure, cow manure vermicompost Sugarcane filter, sugarcane filter cake vermicompost (mixtures)	Not applicable	0.12–0.59	Nourbakhsh (2007)	[29]
11	Municipal solid waste	yard waste (food waste, mixed paper, yard waste, leaves, branches, grass clippings)	0.005–0.1	Komilis (2004)	[56]

## Data Availability

Not applicable.
